# Supplementary material for: A Global Screen for Assembly State Changes of the Mitotic Proteome by SEC-SWATH-MS
Source: Cell Syst. 2020 Feb 26;10(2):133–155.e6. doi: 10.1016/j.cels.2020.01.001 (PMC7042714; doi:10.1016/j.cels.2020.01.001)

Q9NZM1 | MYOF_HUMAN | MYOF FER1L3 KIAA1207
Monomer MW [kDa]: 234.709 Monomer expected elution fraction: 33

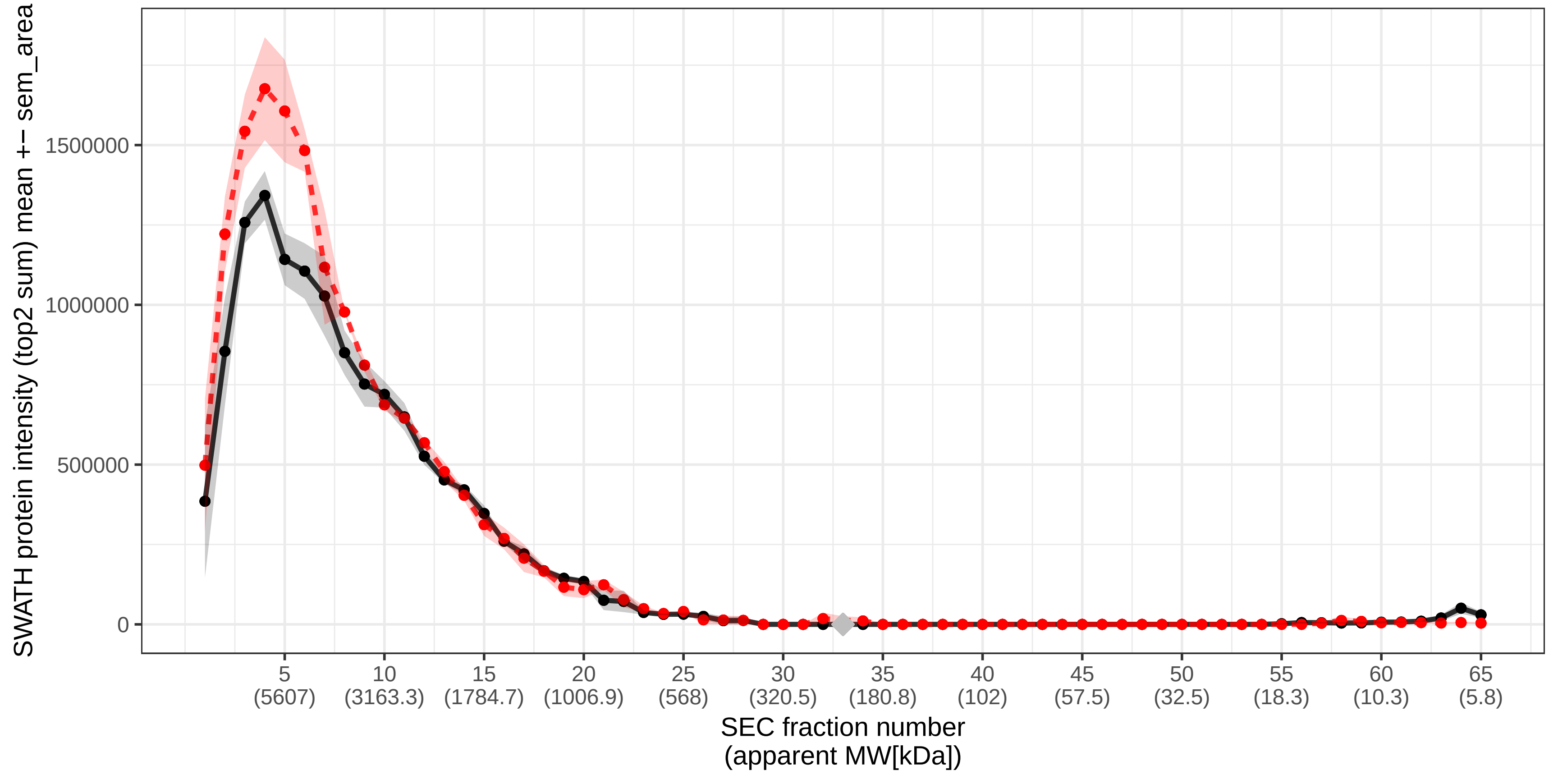

Supplement: Data S1. SEC-SWATH-MS Protein Chromatograms, Related to Figure 1 [file mmc6.zip › SECchrom_Q9NZM1_MYOF_HUMAN_MYOF_FER1L3_KIAA1207.pdf]
